# Preferred mode of delivery and its associated factors in pregnant women with a previous cesarean scar at a tertiary care hospital in Ethiopia: institutional-based cross-sectional study

**DOI:** 10.1186/s12884-023-05891-0

**Published:** 2023-08-15

**Authors:** Abebe Chanie Wagaw, Ashenafi Kibret Sendekie, Solomon Gedlu Nigatu, Getasew Sisay Mihretie

**Affiliations:** 1 Department of Obstetrics and gynecology, School of Medicine, College of Medicne and Health Sciences, University of Gondar, Gondar, Ethiopia; 2 Department of Clinical Pharmacy, School of Pharamcy, College of Medicine and Health Sciences, University of Gondar, Gondar, Ethiopia; 3 Department of Epidemilogy & Biostatistics, Institute of Public Health, College of Medicine and Health Sciences, University of Gondar, Gondar, Ethiopia

**Keywords:** Cesarean scar, Pregnant mothers, Mode of delivery, Trail of level, TOLAC, ERCD, University of Gondar comprehensive specialized hospital

## Abstract

**Background:**

Vaginal births after cesarean or elective repeat cesarean sections (CS) are the options for delivery after one cesarean scar. However, there is a lack of data regarding the preferred next mode of delivery in Ethiopia after a previous cesarean section. Thus, this study assessed the preferred mode of delivery and determinants after one previous CS in the antenatal clinic at the University of Gondar Comprehensive Specialized Hospital (UoGCSH).

**Methods:**

An institutional-based cross-sectional study was conducted among pregnant mothers with one previous CS at UoGCSH from March to August 2022. Structured questionnaires were used to collect the data. The collected data were entered, cleaned, and edited using Epi-data 4.6 and exported to SPSS version 26 for analysis. A binary logistic regression was performed to assess the determinants of the preferred mode of delivery. A p-value of < 0.05 at the 95% confidence level (CI) was considered statistically significant.

**Results:**

The majority, 71.5% (95% CI: 64.7, 77.1), of participants preferred the trial of labor after cesarean (TOLAC) as their mode of delivery. Mothers who were married (AOR = 4.47, 95% CI: 1.19–16.85), had a diploma educational level (AOR = 3.77, 95% CI: 1.84–12.36), had previous post-cesarean complications (AOR = 3.25, 95% CI: 1.08–9.74), and knew about the success of the trial of labor after cesarean (AOR = 13.56, 95% CI: 4.52–37.19) were found to prefer the trial of labor compared with their counterparts.

**Conclusion:**

This study concluded that most pregnant mothers preferred labor trials after one CS, which is a bit lower but comparable with recommended practice guidelines. Providing adequate information and counseling mothers to make informed decisions about their preferred mode of delivery could be substantial.

## Introduction

The introduction of CS has positively impacted maternal and newborn health. However, CS above the World Health Organization (WHO) recommendation of 5–15% may not increase the outcome and prevent maternal and perinatal mortality and morbidity [1]. The decision on the preferred mode of delivery is not easy because of uncertainty and severe complications related to different delivery methods [2]. Lack of certainty and conflicting evidence may impact physician advice about delivery mode after a cesarean scar [3, 4].

A successful labor trial after CS has resulted in decreased costs directly from health expenditures for the mother and family and indirectly from work absentees and welfare losses. It is cheaper than elective repeat cesarean delivery (ERCD) [5]. The trial of labor after cesarean (TOLAC) scars, on the other hand, carries the risk of serious complications such as uterine rupture and perinatal mortality [6, 7]. Previous cesarean scars counts for one-third of CS, and a failed labor trial may result in ERCD [8–10]. Although CS is considered safer than labor trials, it is associated with high rates of maternal complications like abnormal placentation. This limits the number of children, hysterectomies, ICU admissions, longer hospital stays, and neonatal morbidities like temporary respiratory problems [6]. On the other hand, vaginal birth after cesarean (VBAC) is a safe option, but it is dramatically decreasing because of concerns about uterine rupture and perinatal death, despite the fact that these risks are low with less than 1% occurrence [6, 8, 11]. A lack of guidelines, variations in success rates, lack of access to painless delivery, delays in health care services for emergency operations, a lack of proper counseling about options for mode of delivery, unknown scar type, and physicians’ recommendations to have ERCD are factors that declined trial labor [12, 13].

In the developing world, particularly in Ethiopia, TOLAC reduces morbidity and costs associated with ERCD [7]. However, the exact preferred mode of delivery for mothers having previous CS is unknown in this setting and in Ethiopia. As a result, this study determined the prevalence and determinants of delivery methods after CS. Therefore, this study aimed to assess the preferred mode of delivery and its determinants among pregnant mothers with one previous CS scar at the University of Gondar Comprehensive Specialized Hospital (UoGCSH) hospital, Northwest Ethiopia.

## Methods and materials

An institutional-based cross-sectional study was conducted from March 1 to August 30, 2022. The study was conducted at the UoGCSH ANC clinic. The UoGCSH is one of the country’s largest teaching hospitals located in Gondar, Ethiopia. Gondar is one of the country’s historical cities and is 750 km northwest of Addis Ababa. According to 2019 Central Statistics Agency estimates, Gondar City has a population of 500,788 people, with 300,000 males and 200,788 females.

### Study participants and inclusion criteria

The source population was all pregnant mothers with one previous CS scar who visited the UoGCSH ANC clinic. Based on the current and previous clinical and obstetrical characteristics of the mothers, they could be eligible for both TOLAC and ECRD to be included in the study. Pregnant mothers with a gestational age of ≥ 28 weeks who had one prior cesarean scar and attended the UoGCSH ANC clinic were included in this study. Those mothers with multiple gestations, past classical CS scars, and known medical disorders were excluded from this study because they were not candidates for TOLAC.

### Sample size calculation and sampling technique

The single population proportion formula was used to determine the sample size using the following assumptions: a 50% prevalence of either TOLAC or ECRD mode of delivery, a 95% confidence interval, a 5% margin of error, and a 10% non-response rate. Each month, 40 patients with one cesarean scar visited the ANC clinic at the UoGCSH. As a result, during the previous six months of data collection, it was estimated that 240 pregnant mothers with one previous CS scar could have visited the ANC clinic [14].

The required sample size was obtained by the following calculation: n = (za/2) ² p (1-p)/d²,

Where n = sample size, Z = 1.96 (at 95% confidence level), p = proportion of the population, assumed to be 50%, d = margin of error or degree of accuracy desired (0.05), and n = (1.96)2 × (0.50) (0.50)/ (0.05) ^2^ = 384.

The sample was taken from a relatively small population (N = 480; those were mothers with one previous CS scar per monthly record of the study hospital), and since the study population is less than 10,000, the finite population correction formula was used: n = n/ (1 + n/N), n = 384/(1 + 384/480) = 213.3. Taking the 10% expected contingency into account, the final sample size was 235. The study subjects were approached using a simple random sampling technique.

### Operational definitions

Trial of labor after cesarean section (TOLAC): This is an attempt to birth vaginally after a cesarean scar.

Vaginal birth after cesarean (VBAC): The success of labor trial after cesarean scar affecting vaginal delivery.

Elective repeat cesarean after cesarean (ERCD): a cesarean section performed before labor.

Parity: All deliveries that reach viability age (28 weeks in the Ethiopian context).

Gravidity: All pregnancies, regardless of outcome, duration, number, or site.

Gestational age: The age of a pregnancy based on a regular menstrual cycle or early ultrasound estimates.

### Data collection tool and procedure

A structured data collection questionnaire was developed after an extensive review of different literature previously conducted with the same study objectives [8, 15–17] The questionnaire was prepared originally in English and translated into Amharic and back into English by language experts to ensure consistency. The data collection was done using the Amharic version.

The tool was organized into three parts. The first part consisted of socio-demographic characteristics of mothers such as age, education level, residence, marital status, and occupation. The second section of the data collection tool was about previous clinical characteristics related to previous obstetrics. These characteristics included parity, delivery outcome, prior complications, and birth weight. The last section was about issues related to current pregnancy and future preferences for delivery methods. Data was collected from study subjects at the ANC clinic. Participants were interviewed by three trained midwives after written and/or verbal informed consent was obtained from the participants. The data collectors received half-day training regarding the study purpose, data collection tools, and ethical considerations during data collection.

### Data quality management techniques

Before the actual data collection period, the questionnaires were pretested with 5% of the sample size at the Debre Tabor Comprehensive Specialized Hospital ANC clinic. This was done to determine the completeness, ease of use, and reliability of the data collection instrument. Then, some modifications were made. The data was checked for cleanliness and completeness daily after each completed questionnaire. The principal investigator followed the data collection procedure closely and regularly. After data collection was finished, it was checked for completeness and accuracy.

### Data processing, analysis and interpretation

The collected data were entered, cleaned, and edited in EPI-data 4.6 and exported to SPSS version 26 for analysis. The normal distribution of the data was examined with a histogram and Q-Q plot. Mean with standard deviation (SD) was used to describe continuous variables, while frequency with percentage was used for categorical variables. The associations between preferred delivery modes and associated factors were determined by logistic regression analysis. In the univariable association of the preferred mode of delivery and independent variables, variables with a p-value of ≤ 0.20 were further analyzed through multivariable logistic regression to control for confounder effects. A p-value of < 0.05 at a 95% confidence interval was considered statistically significant.

### Ethical approval and consent

The proposal was ethically approved by the ethical review committee of the School of Medicine at the University of Gondar with reference number SOM/1482/2022. After the purpose of the study was explained to the participants, they were in a position to give consent, and they agreed to sign a written consent form. The participants’ involvement in the study was voluntary; participants who were unwilling to participate in the study and those who wished to quit their participation at any stage were informed to do so without any restrictions. Confidentiality was maintained, and the data was sufficiently anonymized at all levels of the study.

## Results

### Socio-demographic characteristics of the participants

A total of 235 participants were involved in the final study. The majority (96.6%) of the participants were married, with a mean age of 30.6 (± 4.2). The majority of participants (96.6%) were city dwellers with a diploma or higher education (Table 1).

**Table 1:** Socio-demographic of mothers with one previous cesarean section at the UoGCSH, Northwest Ethiopia from March to August 2022 (N = 235)

Variables		Frequency (%)	Mean(± SD)
Age			30.6(± 4.2)
Marital status	Married	222(94.5)	
	Divorced	13(5.5)	
Residence	Urban	227(96.6)	
	Rural	8(3.4)	
Educational status	No formal education	23(9.8)	
	Primary level	21 (8.9)	
	Secondary	67(28.5)	
	Diploma	61(26.0)	
	Degree/above	63(26.8)	
Occupation	House wife	113(48.8	
	Government employee	74(31.5)	
	Private employee	32(6.8)	
	Private business owner	16(6.8)	

### Clinical character characteristics of the participants

The most common indication was a non-reassuring fetal heart rate (NRFHRP), reported by 93 (39.9%) of participants. The majority of participants (207, or 88.1%) have live babies, and nearly three-quarters (172, or 73.2%) had previous deliveries weighing between 2500 and 3999 g. Less than a fifth of participants had a post-caesarean complication (39, or 16.6%), and wound infection was the most common (32, or 13.6%) (Table 2).

**Table 2:** Clinical characteristics of the participants and previous obstetrics related factor of mothers with one previous cesarean section at UoGCSH, Northwest Ethiopia, from March to August 2022 (N = 235)

Variables		Frequency (n)	Percentage (%)
Parity	Para one	131	55.5
	Para two	65	27.7
	Para three and above	39	16.6
Previous indication	NRFHRP	87	37
	Malpresentation	40	17
	CPD	16	6.8
	APH	12	5.1
	Failed induction	45	19.1
	Others	35	14.9
Birth weight	< 2500 g	28	11.9
	2500–3999 g	172	73.2
	> 4000 g	53	14.9
Previous outcome	Term alive	207	88.1
	Still birth	9	3.8
	Asphyxiated	9	3.8
	Preterm	10	4.3
Previous Obstetrics and/or medical complication	APH	8	3.4
	HDP	5	2.1
	GDM	5	2.1
	PROM	8	3.4
	NO	209	88.9
Post CS complications	Yes	42	17.9
	No	193	82.1
If yes what was the complication	Excessive bleeding	3	1.3
	Wound infection	32	13.6
	Postoperative pain and numbness	2	0.9
	Delaying in resuming activity	2	0.9

### Current pregnancy and future preference of mode of delivery

**Fig. 1:**
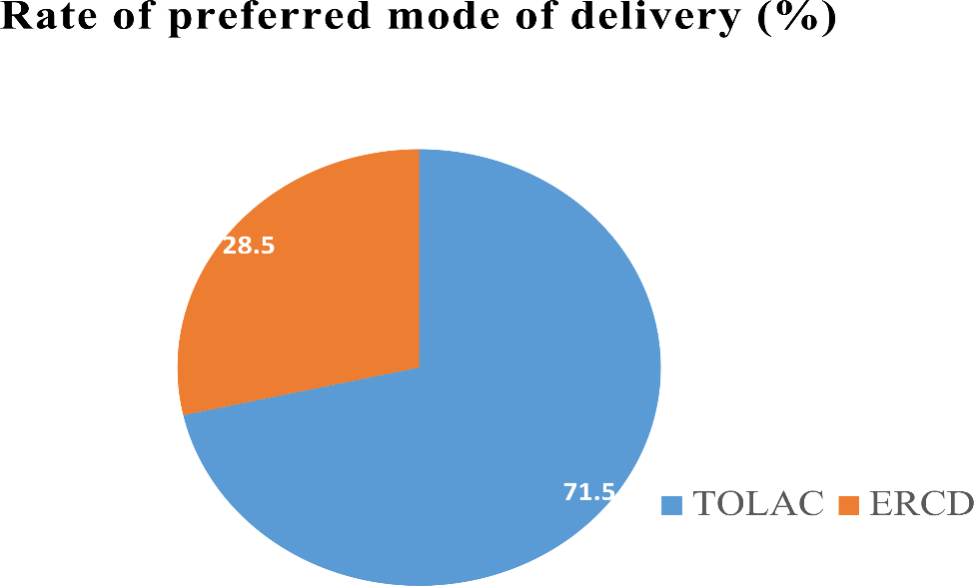
Proportion of preferred mode of delivery of mothers with one previous cesarean section at the UoGCSH, Northwest Ethiopia from March to August 2022 (N = 235)

The majority, 168(71.5%: 95% CI (64.7–77.1)), of the participants preferred TOLAC, as illustrated in Fig. 1. The mean (± SD) estimated gestational age of participants was 33.05 (± 3.9) weeks. The most reported reasons for preference of TOLAC were that nearly one-fourth (23.4%) of the participants considered TOLAC as the preferred mode of delivery to avoid surgery and anesthesia complications, seek early recovery and resumption of activities, and that it is a natural route. The majority of participants (85.5%) knew about TOLAC’s success. TOLAC complications were known to 36.6% and 33.6% of participants, respectively, as uterine rupture and birth asphyxia from a ruptured uterus (Table 3).

**Table 3:** Clinical characteristics related to the current pregnancy mothers with one previous cesarean section at the UoGCSH, Northwest Ethiopia from March to August 2022 (N = 235)

Variables		Frequency (%)	Mean(± SD)
Gestational age	Preterm	176(74.9)	33.05(3.9)
	Term	59(25.1)	
Antenatal counseling provided by	Doctors	109(46.4)	
	Midwives	52(22.1)	
	Family member	11(4.7)	
	Doctors and midwives	26(11.1)	
	Not given	7(3.0)	
	Midwife and family	9(3.8)	
	Doctors and family	8(3.4)	
	Doctors’ family and midwives	13(5.5)	
Why you preferred ERCD	Fear of labor pain	28(11.9)	
	Fear of uterine rupture	10(4.3)	
	Fear of perinatal death	2(0.9)	
	It’s my choice	5(2.1)	
	Fear of labor pain, my choice	3(1.3)	
	Fear of birth asphyxia	3(1.3)	
	Fear of labor pain and uterine rupture	14(6.0)	
	Fear of labor and birth asphyxia	2(0.9)	
Advantage of ERCD over TOLAC	Scheduled	3(1.3)	
	Low risk of uterine rupture	8(3.4)	
	Avoids labor pain	43(18.3)	
	Avoids birth asphyxia	3(1.3)	
	Scheduled and low risk of uterine rupture	4(1.7)	
	Avoids labor pain and birth asphyxia	6(2.6)	
Why TOLAC is preferred	Natural route	25(10.6)	
	Need labor experience	2(0.9)	
	Need to have more children	8(3.4)	
	To avoid surgery and anesthesia complication	6(2.6)	
	Need to have more children, avoid surgery and anesthesia complication, early activity(recovery)and discharge	14(6.0)	
	Natural route and to have more child	4(1.7)	
	early activity (recovery)and discharge	8(3.4)	
	avoid surgery and anesthesia complication, early activity(recovery)and discharge	46(19.6)	
	avoid surgery and anesthesia complication, natural route	55(23.4)	
What complication of TOLAC do you know?	Uterine rupture	86(36.6)	
	Perinatal death from ruptured uterus	8(3.4)	
	Perinatal asphyxia from ruptured uterus	79(33.6)	
	May end up in emergency CS	18(7.7)	
	I never know	32(13.6)	
	Uterine rupture, Perinatal asphyxia and death from ruptured uterus	10(4.3)	
	Perinatal asphyxia and death from ruptured uterus	2(0.9)	
Do you think TOLAC could be successful	Yes	201(85.5)	
	No	21(8.9)	
	I don’t know	13(5.5)	
Who guide you the decision	Doctors	148(63.0)	
	Midwives	54(23.0)	
	Family member	9(3.8)	
	Not given-myself	23(9.8)	
	Doctors, midwives, family	1(0.4)	
ANC visit before	UoGCSH	195(83.0)	
	Health centers	22(9.4)	
	Private visit	18(7.7)	

### Associated factors of mode of delivery

Variables potentially associated with the preferred delivery method were identified using logistic regression analysis. Then, after running into multivariable logistic regression analysis, it showed that only marital status, educational status, previous post-cesarean complications, and knowing the success of TOLAC were found to have an independently significant association with a preference of mode for delivery in the study participants.

Consequently, married women preferred TOLAC 4.47 times higher than divorced ones, with a 95% CI of 1.186–16.848. Those with a diploma level were more likely to prefer TOLAC than those with a degree and above (AOR = 3.773, with a 95% CI of 1.844–12.356). Similarly, participants with post-cesarean complications preferred TOLAC (AOR = 3.247, 95% CI: 1.082–9.741) compared with their counterparts. Moreover, those participants who knew about TOLAC success preferred to have it more frequently than those who did not know (AOR = 13.359, 95% C1: 4.520–37.189) (Table 4).

**Table 4:** Univariable and multivariable association of mode of delivery and other variables

Variables		Mode of delivery		95% CI		P-value
		TOLAC	ERCD	COR	AOR	
Marital status	Married	163	59	4.420(1.391–14.049)	4.471(1.186–16.848)	0.027*
	Divorced	5	8	1	1	
Educational status	No formal education	18	5	1.864(0.647–5.374)	1.728(0.410–7.283)	0.005*
	Primary	12	9	3.947(0.813–19.159)	1.228(0.371–4.062)	
	Secondary	51	16	1.598(0.781–3.270)	2.038(0.873–4.754)	
	Diploma	50	11	2.520(1.146–5.586)	3.773(1.844–12.356)	
	Degree and above	37	26	1	1	
Parity	Para one	90	41	0.399(0.155–1.027)	0.406(0.117–1.411)	0.101
	Para two	45	20	0.409(0.148–1.131)	0.329(0.087–1.241)	
	Para three	33	6	1	1	
Indications	NRFHRP	69	18	1.533(0.624–3.765)	1.622(0.589–4.468)	0.214
	Malpresentation	30	10	1.200(0.431–3.343)	2.006(0.575–7.002)	
	CPD	7	9	0.311(0.091–1.065)	0.392(0.089–1.719)	
	APH	6	6	0.400(0.104–1.541)	0.195(0.078–1.961)	
	Failed induction	31	14	0.886(0.337–2.331)	0.706(0.233–2.145)	
	Others	25	10	1	1	
Post CS complications	Present	37	5	3.502(1.312–9.346)	3.247(1.082–9.741)	0.036*
	No	131	62	1	1	
Gestational age	Preterm	122	54	0.638(0.319–1.278)	0.678(0.294–1.563)	0.362
	Term	46	13	1	1	
Do you think TOLAC will be successful?	Yes	154	47	8.191(3.009–23.300)	13.359(4.520-37.189)	< 0.001*
	I don’t know	8	5	4.000(0.925–17.302)	7.417(0.979–43.880)	
	No	6	15	1	1	

**Others**; cord prolapse, twin pregnancy, oligohydramnios; * indicates a p-value < 0.05.

## Discussion

According to the investigators’ knowledge and searches, the prevalence of preferred modes of delivery and the factors associated with them have not yet been studied in Ethiopia. This is particularly true in the study area. Therefore, assessing the preferred mode of delivery after previous CS scarring and its associates was essential to understanding the real practice and perception of pregnant mothers when it comes to delivery. Accordingly, this study revealed that less than three-fourths of the study participants favored TOLAC as their mode of delivery. Sociodemographic and clinical characteristics such as marital status, educational background, information on TOLAC success, and previous CS complications experience were found to have a significant association with their preferred mode of delivery.

The present study revealed that TOLAC was the most common preferred mode of delivery among pregnant mothers who had one CS scar, with a proportion of 71.5% (95% CI: 64.1–77.1). This finding is a bit lower, but it is comparable with practice guidelines, which recommend TOLAC as a mode of preference for approximately 80% of mothers after one CS scar [18]. Consistent with the current study, a cross-sectional study conducted in Nigeria also showed that TOLAC was the preferred mode of delivery after CS at 73.5% [19]. Another prospective observational study conducted in Japan confirmed that the prevalence of TOLAC as the preferred mode of delivery was 64.1%, which is consistent with this study [20]. Additional research from various countries and institutions revealed that the majority of mothers with previous cesarean scars opted for a trial of labor; in the United States, 83.6% of mothers [10], Pakistan, 80.7% of mothers [21], Poland, 73.3% of mothers [22], and Australia [23], the majority of mothers also opt for TOLAC. The finding may indicate that the majority of mothers prefer natural labor. This might be due to the fear of complications associated with surgery and anesthesia, the delay in recovery, and the early resumption of routine activities. Therefore, besides the indication of the mode of delivery, the mother’s preference could be accounted for, and shared decision-making is recommended.

In contrast to these findings, studies in the Ireland (39.5%) [16], at the University of Massachusetts (45%) [24], and Peru (59%) [25] revealed that a lower proportion of study participants favored TOLAC. On the other hand, other studies have shown that ECRD was the best delivery method. A cross-sectional prospective study conducted in Jordan confirmed that more than half (55%) of the participants preferred ERCD as their chosen mode of delivery [12]. Another cross-sectional descriptive study in Kenya showed that 67.2% of cases preferred ERCD [26]. There is no consistency in delivery mode preference, as seen in this study and other similar studies conducted in different countries. This might be due to differences in socio-demographic and clinical characteristics and experience related to the previous cesarean section of the study population. This could be due to mothers’ and family members’ knowledge and attitudes, as well as cultural influences regarding the benefits and drawbacks of each mode of delivery. In addition, there could be the fear of medical litigation. Moreover, healthcare providers’ practices and variations in guideline recommendations might have contributed to this discrepancy. The other possible explanation could be the providers’ uncertainty. Moreover, the lack of a high level of established evidence, like randomized controlled trials (RCT), about the comparative outcomes of TOLAC versus ERCD could result in variation in their preferences [27].

The current study also identified significant variables that influence mothers’ preference for delivery mode. Consequently, married gravidas had a higher likelihood of TOLAC than divorced mothers. This finding is consistent with a previous study conducted in Kenya [28]. This finding may implicate the fact that married mothers prefer vaginal delivery because of social support from their partners. In contrast to the current findings, previous research has shown that marital status is not associated with a preference for the birthing method [26, 29].

The results of this study also indicated that diploma educational levels are strongly associated with TOLAC compared with mothers with a degree or higher education. The discovery may implicate mothers with high academic backgrounds who may be hesitant to have a child after a previous scar. This finding correlates with other studies. A descriptive study in Turkey showed that mothers who had university status or an above-average academic level preferred ECRD [30]. A retrospective cohort study conducted in Montreal, Canada, shows that mothers with higher education tend to have CS [31]. Similarly, another conducted by Hossain M.T. et al. also found that increased maternal educational status is associated with an increased preference for cesarean delivery [32]. Moreover, a cross-sectional study done in Nigeria also indicated that an increase in educational status was associated with an increase in preference for ERCD [19]. The findings may help to explain why these higher-achieving mothers are concerned about the possibility of experiencing because it lasts longer, becomes more gradual or intense as it progresses, and involves a large number of muscles, ligaments, nerves, and the skin surfaces. Contrary to this study and other scientific evidence that a lower level of education is associated with an increased cesarean rate, this may be due to low prenatal access in those with a low level of education [33]. Some other studies did not report any significant relationship between women’s cesarean preference and their educational level [34]. The inconsistency in the impact of education level might be due to different levels of understanding of the advantages and disadvantages of each mode of delivery.

This study also disclosed that women with previous post-cesarean complications were more likely prefer TOLAC over ERCD than those without complications. Consistent with this study, a study conducted in the US shows women who need early recovery opt for VBAC [24]. Another study conducted in Japan to investigate women’s preferences found that more women preferred TOLAC due to inadequate anesthesia and their prior experience with post-operative abdominal pain [35]. The findings indicate that once a mother had a previous post-cesarean complication, she preferred TOLAC due to her fear of the risk of recurrence of previous surgically related complications. Generally, a negative birth experience or outcome has an impact on the desired mode of delivery in that the prior negative experience had a significant influence on the choice of mode of delivery [36].

Indeed, this particular study also showed that knowledge of TOLAC’s success increased its preference as a mode of delivery over ERCD. A cross-sectional study conducted in Jordan regarding factors influencing the mode of delivery after CS has shown that those who have adequate information about the pros and cons of TOLAC intended to have TOLAC [37]. A similar study done in the USA showed that mothers who have adequate knowledge of TOLAC are more likely to prefer TOLAC [38]. As a result, this study and other scientific evidence demonstrated that counseling about TOLAC success, including its benefits and drawbacks, as well as information about ERCD, enabled mothers to make informed decisions. In addition to proper counseling, mothers’ understanding and perspectives should be assessed, as patients may change the mode of delivery for different reasons. Because of shared decision-making, only 60–80% of TOLAC patients have successful VBAC. Another issue that contributes to difficulties in selecting a mode of delivery after CS is that women do not understand the advantages and disadvantages of each mode of delivery; this can be attributed to a lack of counseling [9, 35, 39]. Therefore, mothers should be counseled, the development of a decision model is critical, and the information provided should be updated scientifically [12, 40].

In general, this study suggests the importance of addressing the dilemma of birth route preference after a cesarean section during the antenatal period. As many factors influence maternal choice during antenatal care, assessing their preferences and decisions could be crucial. It is important to plan the mode of delivery during the preconception phase. Thus, during ANC, patients with a prior cesarean scar should be counseled on modes of delivery, with pros and cons for each mode of delivery [41]. However, practically, the mode of delivery cannot be optimally determined due to the risk imbalances associated with each mode [26, 42]. As the rate of primary cesarean increases, many women face difficulty deciding on the mode of delivery after one scar. Physicians should provide psychological support in decision-making [21]. The decision on the mode of delivery should be in line with maternal preferences and priorities, with clear discussions of the risks and benefits of each mode of delivery [25]. In recent years, the rate of CS has increased and is on the rise in both low- and high-income countries, and efforts are being made to reduce repeat CS in eligible patients to increase VBAC [24, 35].

### Strength and limitation of the study

The study could not address the actual mode of delivery. The relatively small sample size used in this study resulted in an inflated effect size and a wide range of confidence intervals in some variables. In addition, the findings might be difficult to generalize to all mothers in Ethiopia because a relatively small number of study participants from a single center were studied. Despite the limitations of the study, we hope this study can add knowledge for mothers and healthcare practitioners. It will be a baseline for future research on the Ethiopian population.

## Conclusion and recommendation

The current study concluded that pregnant women preferred TOLAC after one previous CS. Previous maternal post-cesarean experience and information gained during ANC regarding the pros and cons of preferred modes of delivery could impact their choice of delivery. In addition, socio-demographic factors such as marital status and educational background were also found to correlate with their preferences. Therefore, providing adequate information and counseling to mothers to make informed decisions about their mode of delivery could be an important point. A prospective study using a relatively large sample size regarding preferences and actual delivery modes might be valuable for future research considering potential variables associated with modes of delivery.

## Data Availability

All necessary materials are within the manuscript. The datasets generated and/or analyzed during the current study are not publicly available to protect from unnecessary abuse of full data of the participants but are available from the corresponding author on reasonable request.

## Abbreviations

CS: Cesarean section
ERCD: Elective repeat cesaran delivery
TOLAC: Trial of labor after cesarean
UoGCSH: University of Gondar Comprehnsive Specialized Hospital
VBAC: Vaginal birth after cesarean
WHO: World health organization
